# Expression of p53 in the Effects of Artesunate on Induction of Apoptosis and Inhibition of Proliferation in Rat Primary Hepatic Stellate Cells

**DOI:** 10.1371/journal.pone.0026500

**Published:** 2011-10-28

**Authors:** Peng Longxi, Fang Buwu, Wang Yuan, Gao Sinan

**Affiliations:** Department of Pharmacology, Tianjin Medical University, TianJin, China; Health Canada, Canada

## Abstract

**Background:**

Activation of hepatic stellate cells (HSCs) plays an important role in the development of cirrhosis through the increased production of collagen. p53, the “guardian of the genome”, is a transcription factor that can bind to promoter regions of hundreds of genes where it either activates or suppresses gene expression. Thereby, p53 serves as a tumor suppressor by inducing cell cycle arrest, apoptosis, senescence and DNA repair. Artesunate is a derivative of Artemisinin, Scholars had found it had more extensive pharmacological effects past 10 years. However, little is known about the expression of p53 in the effects of Artesunate on induction of apoptosis and inhibition of proliferation in rat HSCs.

**Methodology/Principal Findings:**

Isolated and cultured rat primary HSCs in the flask for 10 days to make cells activated. HSCs were divided into two groups: experimental groups and control groups, experimental groups included with various concentrations of Artesunate (125, 150, 175, 200, 225 µmol/L) for 24, 48 and 72 hours. Analysis of MTT revealed that activated HSCs treated with various concentrations of Artesunate (150–225 µmol/L) were inhibited on dose and time-effect relationships; Concentration of hydroxyproline in supernatant was detected by digestive method; Analysis of flow cytometry demonstrated that Artesunate could arrest cell cycle in G1 and induce apoptosis; The nuclear morphological changes in apoptotic cells were evaluated with DNA staining by Hoechst 33258 dye; The expression of p53 were up-regulated showed by western blotting and RT-PCR.

**Conclusion:**

Artesunate could inhibit HSCs proliferation in dose-dependent and time-dependent manners *in vitro* through increase the expression of p53.

## Introduction

Liver fibrosis is a compensatory response to a variety of chronic liver injury, is the hub of the development of “chronic hepatitis - liver fibrosis – cirrhosis”. At present, liver fibrogenesis is considered a dynamic process involving complex cellular and molecular mechanisms, resulting from the chronic activation of the tissue repair mechanisms, which follows recurring liver tissue injury. If the chronic damage persists, inflammation and fibrosis can progress to liver cirrhosis, ultimately leading to organ failure and death [1]. In the process of hepatic fibrosis, activation and proliferation of hepatic stellate cells (HSCs) is the central link in the incidence [2]. Hepatic stellate cells, also known as “Ito cells” or “fat-storing cells”, localize between hepatocytes and sinusoids (space of Disse) in mammalian livers. In their healthy state, HSCs control retinoid homeostasis, sinusoidal blood flow, macromolecule transport, and potentially act as antigen-presenting cells in the liver [3], [4]. However, in response to hepatic injury, HSCs undergo gross morphological and functional changes, transforming to a myofibroblast-like phenotype in a process called “activation” or “trans-differentiation”. Activated HSCs produce a number of profibrotic cytokines and growth factors that perpetuate the fibrotic process through paracrine and autocrine effects [5]. So preventing HSCs activation and promoting HSCs apoptosis is an important measure for prevention and treatment of liver fibrosis.

p53, the “guardian of the genome”, is a transcription factor that can bind to promoter regions of hundreds of genes where it either activates or suppresses gene expression. Thereby, p53 serves as a tumor suppressor by inducing cell cycle arrest, apoptosis, senescence and DNA repair [6]. In normal cells, p53 is frequently undetectable due to fast ubiquitination by mdm-2 and subsequent proteasomal degradation. However, upon DNA damage and several other stresses, including drug stress, the amount of p53 is increased due to disruption of its degradation.

Artesunate is a derivative of Artemisinin. It had more extensive pharmacological effects, such as anti-cancer [7], treatment of lupus erythematosus [8] and anti-parasite [9]. But the standpoint that Artesunate had anti-hepatic fibrosis effect was first proposed by our group. In our previous studies, Artesunate could effectually inhibit liver fibrosis in rats induced by carbon tetrachloride [10] or immuned with bovine serum albumin [11], and proliferation of HSC-T6 cell line [12]. But there has not been reported about the effects of Artesunate on rat primary hepatic stellate cells. To further approach the mechanism of Artesunate on anti-hepatic fibrosis, in this study, we isolated and cultured rat primary HSCs, observed the effects of Artesunate on proliferation, cell cycle, apoptosis in cultured activated HSCs. We found that Artesunate could inhibit HSCs proliferation *in vitro* through increase the expression of p53.

## Results

### Hepatic stellate cells isolation , culture and identification

**Figure 1:**
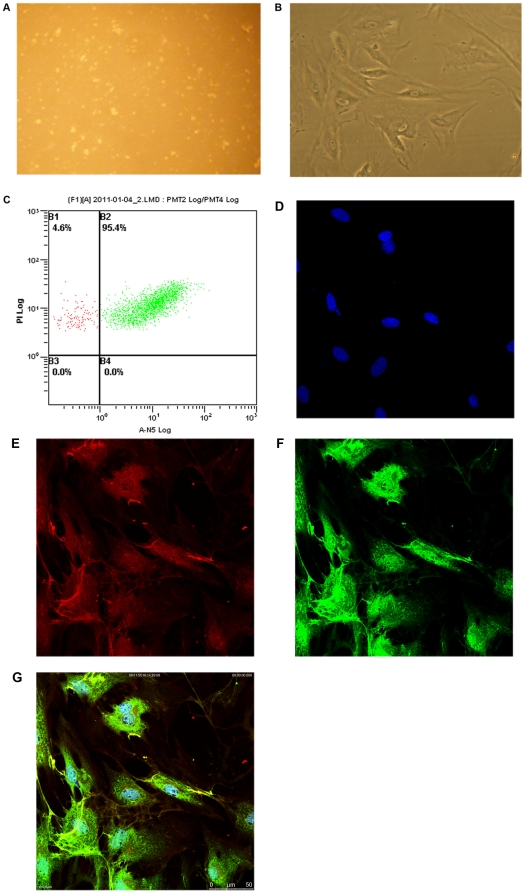
Hepatic stellate cells identification. A. Freshly isolated HSCs were spherical, had strong refraction and suspended in culture medium (×100). B. 10-day primary cultured HSCs were fully spreading, presenting stellate or polygonal forms, granular cells were significantly reduced (×100). C. Cell purity was detected by flow cytometry: freshly isolated HSCs stained by monoclonal anti-desmin and FITC-anti-IgG, and the the positive cells in freshly isolated HSCs were 95.4%. D. Generation 2 HSCs were stained by DAPI: cell nucleus were stained and presented in blue (×600). E. Generation 2 HSCs were stained by GFAP/IgG-TRITC: positive cells were stained and presented in red (×600). F. Generation 2 HSCs were stained by desmin/IgG-FITC: positive cells were stained and presented in green (×600). G. Generation 2 HSCs were stained by GFAP/IgG-TRITC , desmin/IgG-FITC and DAPI: positive cells were stained and the number of HSCs more than 99% (×600).

Freshly isolated rat HSCs were cultured on plastic in serum-containing media. HSCs were observed by phase contrast microscope and immunocytochemistry: HSCs isolated freshly were spherical, had strong refraction and suspended in culture medium (Figure 1A). After culturing for 10 days, HSCs were fully spreading, presenting stellate or polygonal forms, lipid droplet in the cells were significantly reduced (Figure 1B). The yield rate of HSCs was (2–5)×10^7^ per rat liver,and the cell viability was more than 95%. The flow cytometry analysis and the immunocytochemical stain showed that the positive cells in freshly isolated HSCs were 95.4% (Figure 1C) and more than 99% in passage culture (Figure 1D, E, F, G).

### Hepatic stellate cells growth curve

**Figure 2:**
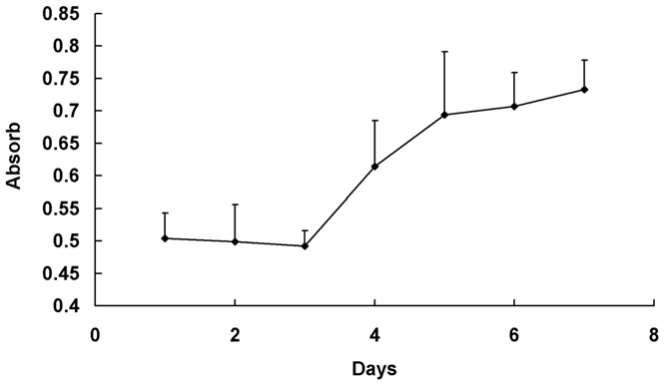
Hepatic stellate cells growth curve. The second generation of HSCs were collected and seeded in 96-well plates by density of 5×10^4^/well, the absorbance was measured with microplate reader ( λ 570 nm), culture time as abscissa, absorbance value as ordinate. HSCs were proliferated after 72 h, turning into the logarithmic growth phase on the 4th, 5th day and into the plateau phase on the 6th, 7th day.

HSCs growth curve showed that the cultured HSCs were proliferated after 72 h, turning into the logarithmic growth phase on the 4th, 5th day and into the plateau phase on the 6th, 7th day (Figure 2).

### Artesunate inhibited HSCs proliferation

Results of MTT colorimetric showed that inhibition of HSCs which cultured with different concentrations of Artesunate for 24, 48, 72 h significantly increased, compared with the control group and the difference was statistically significant (*P*<0.01). In the treatment groups stimulated for 24 h and 48 h, results showed that the inhibitory effect increased with the concentration increasing (*P*<0.01) and suggested that the inhibitory action of Artesunate on HSCs proliferation was in a dose-dependent manner. In group 175 µmol/L, inhibition increased with time increaseing(*P*<0.01), at 72 h, in group 150 µmol/L the inhibition rate reached 92.6%, it suggested that inhibitory action of Artesunate on HSCs proliferation was in a time-dependent manner (Table 1).

**Table 1:** Inhibition rate of Artesunate on HSCs under different concentrations and time (%,
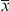
± s).

Group	n	Time (h)		
		24	48	72
control group	6	0.00±0.00	0.00±0.00	0.00±0.00
125 µmol/L	6	6.06±1.44	9.61±4.82	75.59±4.82* &
150 µmol/L	6	21.47±5.57*	26.97±9.28*	92.60±1.18* &
175 µmol/L	6	42.00±7.36* #	70.53±6.77* # ⋆	94.61±0.90* &
200 µmol/L	6	67.12±4.55* △	87.55±2.40* △ ⋆	94.91±4.04*
225 µmol/L	6	79.83±3.67*	94.91±0.99* ⋆	95.32±0.25*

Compared with control group,

*P<0.01; compared with 150 µmol / L,

# *P*<0.01; compared with 175 µmol / L,

△ *P*<0.01; compared with acted for 24 h,

⋆ *P*<0.01; compared with acted for 48 h,

& *P*<0.01. Inhibition of HSCs which cultured with different concentrations of Artesunate for 24, 48, 72 h significantly increased, compared with the control group and the difference was statistically significant (*P*<0.01). In the treatment groups stimulated for 24 h and 48 h, results showed that the inhibitory effect increased with the concentration increasing (*P*<0.01) and suggested that the inhibitory action of Artesunate on HSCs proliferation was in a dose-dependent manner. In group 175 µmol/L, inhibition increased with time increasing (*P*<0.01), at 72 h, in group 150 µmol/L the inhibition rate reached 92.6%, it suggested that inhibitory action of Artesunate on HSCs proliferation was in a time-dependent manner.

### Artesunate decreased the concentration of hydroxyproline in HSCs

After stimulated with Artesunate for 24 h, secretion of hydroxyproline decreased in all experimental groups. Compared with control group, the difference was statistically significant (*P*<0.01) (Table 2).

**Table 2:** Effects of Artesunate on secretion of hydroxyproline of HSCs (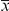
± s).

group	N	Hydroxyproline content in supernatant (µg/mL)
control group	6	1.715±0.030
150 µmol/L	6	1.619±0.026*
175 µmol/L	6	1.515±0.020*
200 µmol/L	6	1.339±0.030*

Compared with control group,

*P<0.01.

### Artesunate induced G1 phase cell cycle arrest in HSCs

**Figure 3:**
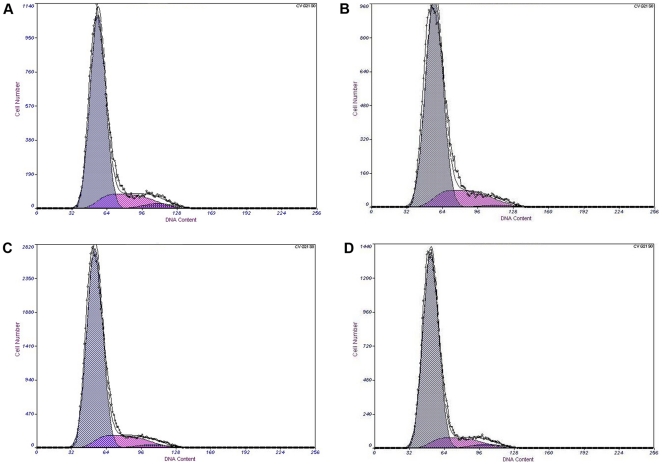
Effect of Artesunate on cell cycle of HSCs. A: control group; B: 150 µmol/L Artesunate group; C: 175 µmol/L Artesunate group; D: 200 µmol/L Artesunate group. In groups 175, 200 µmol/L, the percentage of cells at G0/G1 phase increased significantly (*P*<0.01) , compared with the control group, the percentage of S phase decreased significantly (*P*<0.05), G2 / M phase cells did not obviously change.

In 175, 200 µmol/L groups, the percentage of cells at G0/G1 phase increased significantly (*P*<0.01), compared with the control group, the percentage of S phase decreased significantly(*P*<0.05), G2 / M phase cells did not obviously change (Table 3 and Figure 3).

**Table 3:** Effects of Artesunate on cell cycle of HSCs (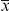
± s).

group	n	G0/G1	S	G2/M
control group	3	78.48±0.44	17.94±0.11	3.58±0.42
150 µmol/L	3	79.06±1.03	19.23±0.80	1.71±0.75
175 µmol/L	3	82.09±0.06*	15.36±0.34^#^	2.55±0.31
200 µmol/L	3	83.16±0.56*	13.62±1.95*	3.21±1.66

Compared with control group, **P<0.01,

*P<0.05.

### Artesunate induced apoptosis in HSCs

**Figure 4:**
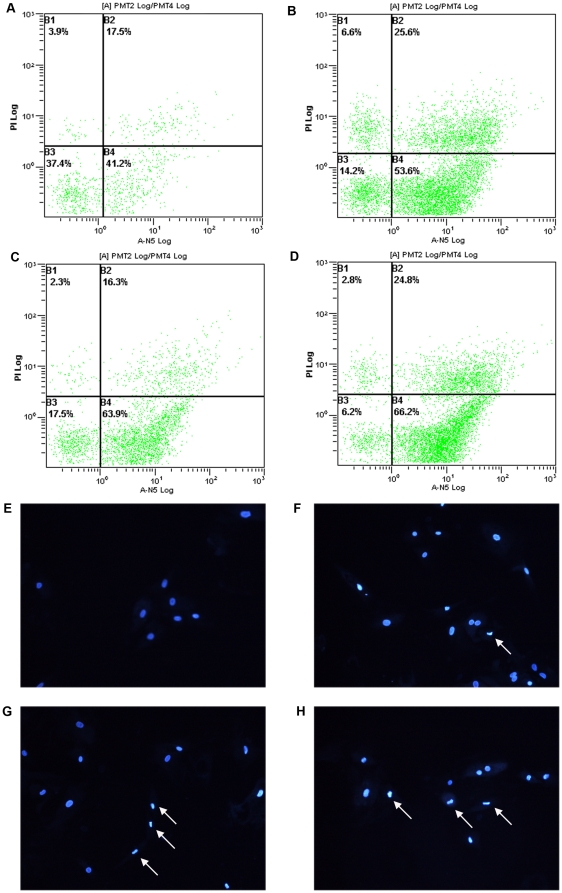
Effects of different concentrations of Artesunate on HSCs apoptosis. A–D: Flow cytometric analysis: Apoptotic index increased with the increasing concentration of Artesunate, apoptosis rate in the control group was (40.73±0.81)%, apoptosis rates of treatment group (artesunate concentrations were 150, 175, 200 µmol / L) were (52.63±0.84)%, (63.97±0.50)%, (66.65±0.99)%, the difference was statistically significant (*P*<0.01). However, HSCs have spontaneous apoptosis without the presence of artesunate. E–H: Hoechst 33258 staining showed apoptosis was induced after Artesunate treatment. In Artesunate groups, nuclei appeared typical morphological changes of apoptosis, some appear condensed chromatin state, a high degree of nuclear chromatin condensation, marginalization, and even serious cleavage fragments, resulting in apoptotic bodies, and the nuclei of the normal control group without significant morphological changes, the nucleus shape rules, to issue uniform fluorescent. A, E: control group; B, F: 150 µmol/L Artesunate group; C, G: 175 µmol/L Artesunate group; D, H: 200 µmol/L Artesunate group.

The results showed that the apoptotic HSCs induced by Artesunate grew in number as the concentration increased. Apoptotic rate of control group was (40.73±0.81) %, rate of treatment groups (concentration of Artesunate were 150, 175, 200 µmol/L, respectively) was (52.63±0.84)%, (63.97±0.50)%, (66.65±0.99)%. Compared with control group, the difference was statistically significant (*P*<0.01) (Table 4 and Figure 4). Apoptotic index increased with the increasing concentration of Artesunate, and HSCs apoptosis induced by Artesunate exceeded spontaneous apoptosis of HSCs.

**Table 4:** Effect of different concentrations of Artesunate on HSCs apoptosis (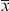
± s, n = 3).

group	apoptosis rate(%)
control group	40.73±0.81
150 µmol/L	52.63±0.84*
175 µmol/L	63.97±0.50*
200 µmol/L	66.65±0.99*

*P<0.01.

### Hoechst 33258 staining

Treatment with Artesunate, HSCs produced Hoechst-positive staining of condensed nuclei that indicated apoptosis of the cells. Significant increase in Hoechst staining was observed among apoptotic HSCs treated with 175 µmol/L Artesunate (Figure 4), which further indicated that Artesunate induced cell apoptosis.

### Artesunate increased protein and mRNA levels of p53 in HSCs

**Figure 5:**
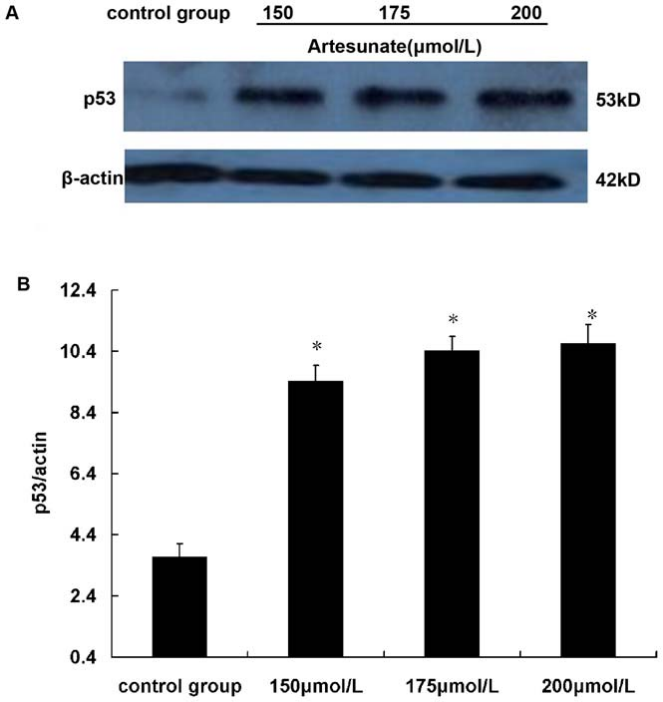
Effects of Artesunate on p53 contents in HSC (n = 6). After HSCs acted by Artesunate 150 µmol/L, 175 µmol/L, 200 µmol/L, Compared with control group, the expression of p53 protein increased significantly in Artesunate group, * *P*<0.05.

**Figure 6:**
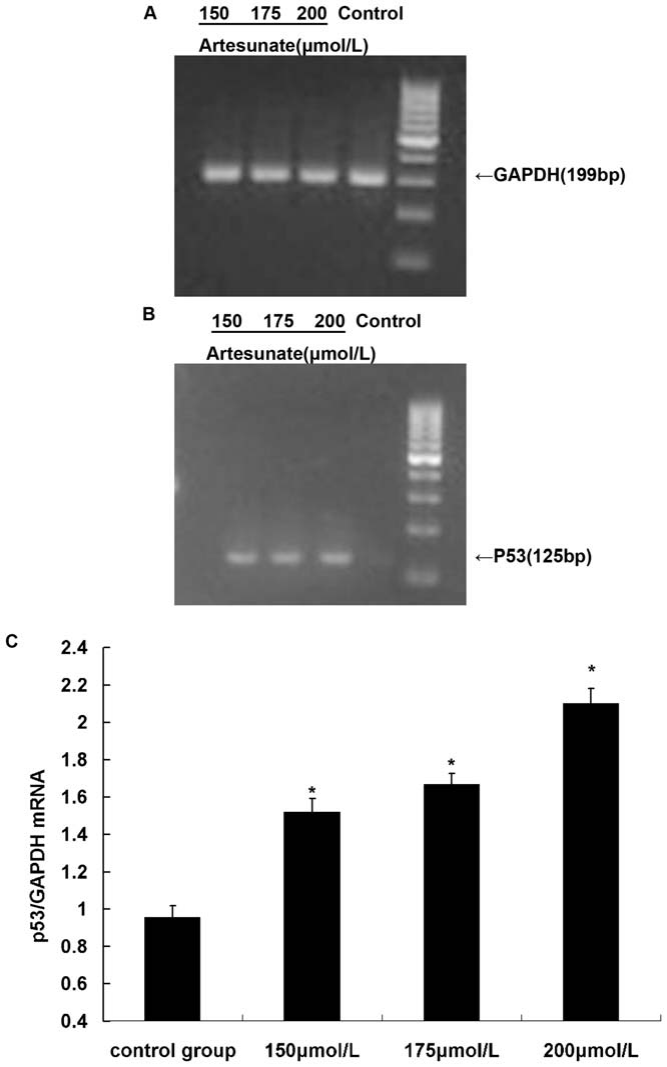
Effects of Artesunate on P53 mRNA expression (n = 6). After HSCs acted by Artesunate 150 µmol/L, 175 µmol/L, 200 µmol/L, Compared with control group, the expression of P53 mRNA increased significantly in Artesunate group, * *P*<0.05.

After HSCs acted by Artesunate 150, 175, 200 µmol/L, respectively, for 24 h, western blotting and RT-PCR showed that p53 was significantly increased, and with the increasing of drug concentration (Figure 5, Figure 6).

## Discussion

Hepatic stellate cells (HSCs), also known as Ito cells or fat-storing cells, are mesenchymal cells mainly in the Disse space, scattering the sinusoid wall. In normal liver, HSCs proliferate at a very low rate, produce a small amount of extracellular matrix (ECM) to maintain homeostasis of ECM production and degradation. During the chronic liver injury, HSCs undergo a process of transdifferentiation from a resting, fat-storing phenotype to myofibroblasts which have a strong ability of proliferation, and produce a large amount of ECM. The activated HSCs are major source of hepatic ECM. Therefore, inhibition of HSCs proliferation and promotion of HSCs apoptosis are currently considered to be effective treatment preventing the development of liver fibrosis in chronic liver disease [13].

HSCs have some features of muscle cells, which contain desmin in cytoplasm, and desmin stably present in the primary and passaged HSCs. However, in normal rat liver, 50 percent of HSCs around the central vein do not express desmin, and HSCs near portal vein have high desmin staining positive rate. Thus, there will be false negative if only desmin used as identification index. Neubauer et al [14] found that glial fibrillary acidic protein (GFAP) expressed in astrocytes was also expressed in HSCs. Except the narrow area around periportal space, GFAP-positive HSCs evenly distributed throughout the hepatic lobule, basically complementary to the distribution of desmin. Therefore, in this study, anti-desmin / FITC, anti-GFAP / TRITC, DAPI stain was used to label desmin, GFAP and nuclei to improve the identification accuracy. And HSCs can be distinguish from the other cells in cell morphology, HSCs were larger, presents a multiple corniform and typical star-shape pattern. In this study, the result of identification showed that the purity of second generation of HSCs has reached 99%. Artesunate is a partially-synthetic derivative of artemisinin, which is extracted from the Chinese herb *Artemisia annua*. Artesunate which has good water solubility and high antimalarial activity is an important derivative of artemisinin. Scholars found it had more extensive pharmacological effects past 10 years, such as anti-cancer, treatment of lupus erythematosus, anti-parasite, anti-endotoxin and treatment of skin diseases. From our previous experimental results, it can be seen that Artesunate can inhibit proliferation of HSC-T6 cell line in 12.5 µg/mL–100 µg/mL, Artesunate (9.6 mg/kg, 28.8 mg/kg, 15 mg/kg orally) can significantly improve rat liver fibrosis induced by CCl_4_ and bovine serum albumin. So in this study, on the basis of the effects of Artesunate on anti-hepatic fibrosis *in vivo* and *vitro*, further investigating the effects of Artesunate on primary cultured HSCs was completed. In this experiment, the MTT test applied to explore the effective dose of artesunate on HSCs. Beginning dose range from 100 mmol/L–100 µmol/L, step by step to narrow dose range. Finally, we found that the sensitivity of primary HSCs to artesunate is low. Results given by our experiments showed that the inhibition of HSCs stimulated with 125 µmol/L Artesunate for 24 h rate was only 6.06%, while the Artesunate concentration to 225 µmol/L, the inhibition rate reached 79.83%. Through repeated tests and IC50 calculation, ultimately, we identified 175 µmol/L as a middle concentration, 150 µmol/L and 200 µmol/L respectively as a low and high concentration. The concentrations we chose were lower than the concentration tested in vivo, we believe that this concentration is feasible. However, in the latter part of the experiments, we found that the differences between the three doses were constantly not significant statistically, it may be due to the interval between three doses were too small. In this study, Artesunate could significantly inhibit proliferation of activated HSCs *in vitro*, and showed dose-effect and time-effect relationships in 150–225 µmol/L. At the same time, Artesunate decreased hydroxyproline in the supernatant, which suggested that Artesunate decrease production/ secretion of collagen.

We use p53 as a starting point in order to further explore the mechanism of Artesunate inhibiting proliferation of HSCs from the cell apoptosis and cell cycle. Apoptosis, or programmed cell death is an active process of cell death controlled by genes to regulate the body development, maintain homeostasis in multi-cell organisms. Apoptosis is physiological regulatory mechanism of internal environment, ensures the normal development of the body by removing unnecessary or harmful cells by apoptosis, and controls the number of cells of tissues and organs together with cell division and proliferation. The previous study found that both mitosis and apoptosis of HSCs were increased in liver fibrosis process, especially proliferation. In reversal phase, apoptosis was prevalent, and HSCs lost net increase. Apoptosis increased collagenase activity, and promoted the degradation of ECM which was conducive to the recovery of liver fibrosis. The results showed that Artesunate could induce apoptosis of HSCs in a dose dependent manner. After stimulating with different concentrations of Artesunate for 24 h, Annexin V positive cells and Hoechst-positive staining cells began to increase. The number of apoptotic cells increased with increasing concentration of Artesunate. Compared with the control group, the apoptosis rate of the Artesunate groups (150, 175, 200 µmol/L) was significant (*P*<0.01). It suggests one of key links of Artesunate anti-fibrosis is induced apoptosis of HSCs, because apoptosis of the activated HSCs means elimination of main source of ECM [15]. HSCs had to be given to other laboratory for flow cytometry after marking Annexin V and PI, as the delay on the way, there were more cells appearing spontaneous HSCs apoptosis from the experimental result. However, the overall trend of drug-induced apoptosis has not changed, compared with the control group, there was significant difference.

Program control of cell cycle is achieved mainly through orderly phosphorylated and dephosphorylated of a variety of cell cycle proteins (cyclin) and cyclin-dependent kinase (CDK) to control activity of cyclin-CDK complex. The cellular transcription factor p53 is involved in “growth arrest” and apoptosis [16]. It shows its effects via repression or activation of other downstream genes and is stimulated by either DNA damage or cellular stress events [17]. In a cell cycle, regulatory function of p53 is presented mainly in monitoring calibration point of G1 and G2 / M phase, and is closely related to transcriptional activation. Many studies had reported the role of p53 protein in inhibition of cell proliferation, such as p53 could induce arrest of cell cycle at G1 phase by acting on p21 [18], p53 could arrest cell cycle at G1 phase by inhibiting myc expression [19]. p53 was the “monitor” of cell growth, an important negative regulator of cell growth. In this study, it is confirmed that Artesunate could raise the level of p53 protein and P53 mRNA, resulting in cell arrest and apoptosis [20]. Flow cytometry showed that after treatment with Artesunate, there were changes in the cell cycle: G0 / G1 phase cells increased, S phase cells decreased, G2 / M phase cells was almost the same, it also confirmed this view.

Overall, it was approved in our study that Artesunate could directly inhibit the proliferation of primary HSCs by increasing the expression of P53 mRNA and p53 protein, resulting in G1 arrest and apoptosis. Artesunate plays an important role in anti-liver fibrosis by inhibiting proliferation and promoting apoptosis of HSCs to decrease production/secretion of collagen. It suggests that Artesunate could be expected to be an ingredient of Chinese medicine with anti-hepatic fibrosis effect.

## Materials and Methods

### Drugs and reagents

1. Drugs: Artesunate (C_19_H_28_O_8_), relative molecular mass 384.42, provided by Guilin South Pharmaceutical Company Limited (Guilin, Guangxi, CHN).
2. Main reagents: Dulbecco's modified Eagle's medium supplemented with 10% foetal bovine serum and antibiotics (penicillin, 100 U/mL; streptomycin, 100 µg/mL) from Hyclone (Thermo, Beijing, CHN). Pronase E were purchased from Roche Diagnostics GmbH (Roche, Mannheim, GER), type IV collagenase were purchased from Sigma (St. Louis, MO, USA). Mouse antibody for human glial fibrillary acidic protein (GFAP), goat antibody for mouse IgG-FITC, rabbit antibody for rat desmin, goat antibody for rabbit IgG-TRITC was purchased from Wuhan Boster Bio-engineering Limited Company (Boster, Wuhan, CHN), hydroxyproline assay kit purchased from Nanjing Jiancheng Biological Engineering Institute(Jiancheng, Nanjing, CHN). Nuclear protein and cytoplasmic protein extraction kit, high sensitivity ECL chemiluminescence kit, HRP-labeled goat anti-mouse IgG (H+L), β-actin antibody, Annexin V-FITC/PI kits and Beyozol were purchased from Beyotime Institute of Biotechnology (Beyotime, Haimen, CHN). Hoechst 33258 was purchased from KeyGEN Biotech(KeyGEN, Nanjing, China). p53 monoclonal antibody was purchased from Invitrogen Corporation (Invitrogen, CA, USA). TransScript First-Strand cDNA Synthesis SuperMix was purchaseed from TransGen Biotech (Trans, Beijing, CHN). GeneRuler™ 100 bp DNA Ladder and DreamTaq™ Green PCR Master Mix were purchased from Fermentas Company(Fermentas, Shenzhen, CHN). Primers P53 and PDGF were synthetized by Sangon Biotech (Sangon, Shanghai, CHN).

### Ethics Statement

All experimental protocols were approved by the Committee for the Care and Use of Laboratory Animals of Tianjin, the permit numbers is TJMU2009001.

### Hepatic stellate cells isolation and culture

This method was improved on the basic method in reference to Friedman [21]. Wistar rats of SPF grade were provided by Laboratory Animal Center of the fourth institute of Military Medical Science Academy of the PLA (Tianjin, China) and maintained under 12∶12-h light/dark cycles with food and water ad libitum. Wistar rats of 300–400 g body weight were poodled and disinfected with 75% (V∶V) alcohol bath, ultraviolet irradiation for 20 min, then ip heparin 50 U after intraperitoneal injection of 25% urethane anesthesia(0.5 ml/100 g); HSCs were isolated by sequential *in situ* perfusion with calcium-free perfusate (142 mmol/L NaCl, 6.7 mmol/L KCl, 10 mmol/L HEPES, 5.5 mmol/L NaOH, pH 7.4) with heparin 4 U/mL, then dissociation of integrated liver to Petri dish, ex vivo perfusion with 50 ml 0.8 mg/mL pronase E, then circulating perfusion for 20 min with 50 ml 0.5 mg/mL type IV collagenase (prepared with perfusate: 67 mmol/L NaCl, 6.7 mmol/L KCl, 5 mmol/L CaCl_2_, 100 mmol/L HEPES, 66 mmol/L NaOH, pH 7.6); the cell suspension was centrifuged by discontinuity density gradient of 18%, 12% and 8% Nycodenz (1450 g/min, 20 min, 4°C). Taking HSCs at 8% and 12% of the gradient interface, adjusting the cell density to 5×10^5^/mL, seeding in culture flasks. Cells were grown in Dulbecco's modified Eagle's medium supplemented with 10% fetal bovine serum with antibiotic. Culture medium was replaced at day 2 after plating and then every 2–3 days. Cells were kept in culture at 37°C in a 5% CO_2_ atmosphere and 100% humidity. For all experiments, passages 2–4 of HSCs were used.

### Hepatic stellate cells characterization

Morphology and growth characteristics of freshly isolated cells were observed with inverted phase contrast microscope. The purity of freshly isolated HSCs were analysed with flow cytometry which all cells were stained by propidium iodide and HSCs were stained by monoclonal anti-desmin / FITC-anti-IgG. The second generation HSCs were stained respectively by anti-desmin / TRITC- anti-IgG, monoclonal anti-GFAP/FITC-anti-IgG and DAPI to identify the HSCs.

### Hepatic stellate cells growth curve drawing

The second generation of HSCs were collected and seeded in 96-well plates by density of 5×10^4^/well. Incubated at 37°C, 5% CO_2_ for 24, 48, 72, 96, 120, 144, 168 hours respectively. Then 10 µl MTT was added in each well, further incubated for 4 h. 150 µl DMSO was added in each well, absorbance (*A*) was measured at λ 570 nm on a microplate reader after a 10 min vibrating. Absorbance in well with only added medium served as control to zero setting. Growth curve was plotted, with culture time as abscissa, absorbance value as ordinate.

### MTT Assay for Cell Proliferation

The effects of Artesunate on cell proliferation were determined by 3-(4,5-dimethylthiazol-2-yl)-2,5-diphenyltetrazolium bromide (MTT) assay. 3×10^3^ cells/well were plated in 96-well culture plates, cultured for 24 h, the cells were treated with various concentrations of Artesunate (125, 150, 175, 200, 225 µmol/L) for 24, 48, 72 h. Control group (had been treated with vehicle only) and each concentration group with six duplicate wells were set up, the absorbance was measured with microplate reader ( λ 570 nm). Inhibition rate by the below formula (inhibition rate, IR) was calculated, inhibition rate = [(*A* value of control group−*A* value of experimental group) / *A* value of control group]×100%.

### Hydroxyproline test in the cell culture supernatant

HSCs at logarithmic growth were seeded in 6-well plates by density of 5×10^5^/mL. Treatment groups (respectively 150, 175, 200 µmol/L) and control group were set up. Culture supernatant was collected after 24 hours, in which hydroxyproline was tested according to instructions of the hydroxyproline assay kit.

### Cell cycle analysis

HSCs at logarithmic growth were seeded in 6-well plates by density of 5×10^5^/mL. Treatment groups(respectively 150, 175, 200 µmol/L) and control group were set up and cultured for 24 hours. HSCs were trypsinized, centrifuged (1000 r/min, 5 min), washed with cold PBS, fixed with cold 70% ethanol/30% phosphate-buffered saline at 4°C overnight. HSCs were then digested by 1000 U RNase A, and stained with 1% propidium iodide at 37°C for 30 min. The DNA profiles were determined within 4 h of staining by flow cytometry (Epcis ALTRA, BECKMAN COULTER, USA). Data were analysed by EXPO32 (BECKMAN COULTER, USA). Fifteen thousand cells were counted under each condition.

### Effects of Artesunate on Hepatic stellate cells apoptosis

HSCs at logarithmic growth phase were seeded in culture flasks by density of 1.5×10^5^/mL. Treatment groups (respectively concentration of 150, 175, 200 µmol/L) and control group were set up. Cells were collected after culturing for 24 h and detected by flow cytometry (Epcis ALTRA, BECKMAN COULTER, USA) according to the instructions of Annexin V FITC / PI kit. To detect early apoptotic changes, staining with annexinV–fluorescein isothiocyanate (FITC) was used, because of its known high affinity to phosphatidylserine.

Phosphatidylserine is normally situated on the inner layer of the plasma membrane. In the course of cell death, phosphatidylserine is translocated to the outer layer of the membrane, this occurs in the early phases of apoptosis, while the cell membrane itself remains intact. In contrast to apoptosis, necrosis is accompanied by loss of cell membrane integrity and leakage of cellular constituents into the environment. To distinguish apoptosis and necrosis, propidium iodide, a common dye exclusion test, and annexin V-FITC were used in parallel to show membrane integrity after annexin V-FITC binding to cells.

### Hoechst 33258 staining

HSCs at logarithmic growth were seeded in 96-well plates by density of 1×10^4^/mL. Treatment groups (respectively 150, 175, 200 µmol/L) and control group were set up and cultured for 24 hours. HSCs were washed twice with PBS, then incubated with 2 µmol/L Hoechst 33258 for 15 min in dark at room temperature. HSCs were viewed under a fluorescence microscope (Nikon, Tokyo, Japan) equipped with a UV filter. The images were recorded on a computer with a digital camera (DXM 1200, Nikon) attached to the microscope, and the images were processed by computer. The Hoechst reagent was taken up by the nuclei of the cells, and apoptotic cells exhibited a bright blue fluorescence.

### Western blotting

HSCs at logarithmic growth phase were seeded in 6-well plates by the density of 5×10^5^/mL. Treatment groups (respectively concentration of 150, 175, 200 µmol/L) and control group were set up. After culturing for 24 hours, cells were washed twice with PBS, lysed by nuclear protein and cytoplasmic protein extraction kit, and then nuclear protein content of the sample was determinated by BCA protein determination kit. After quantification, 20 µg of nuclear protein was taken, separated with 12% sodium dodecyl sulfate-polyacrylamide gel electrophoresis (SDS-PAGE). Protein separation was accomplished by 80 V for 2–3 h. Gels were then transferred at 120 mA for 1.5 h at room temperature onto polyvinylidene fluoride membranes (Immobilon-P^SQ^, Millipore Corporation, MA, USA). Membranes were blocked in blocking buffer consisting of 5% non-fat milk in TBS-T (Tris-buffered saline Tween-20). Primary antibodies against p53 diluted in blocking buffer at 1∶ 1000 were incubated with the above PVDF membrane transferred by the proteins in gel overnight at 4°C, followed by three washes. The β-actin antibody as loading control was diluted at 1∶ 2000 overnight at 4°C also. Membranes were incubated with horseradish peroxidase-conjugated secondary antibodies for 2 h and then washed. Membranes were colored with ultra-sensitive ECL chemiluminescence kit and film was exposured. After developing and fixing, figure was scanned. Densitometric evaluation of the blots was performed using the program Image J.

### Reverse-transcription PCR

Levels of mRNA for P53 were determined by RT-PCR. Total RNA was isolated from cells using the Beyozol. cDNA was synthesized according to Reverse Transcription kit manufacturer's instructions, using the following program: reverse transcription at 42°C for 30 min, 85°C for 5 min, and then cDNA was amplified by DreamTaq™ Green PCR Master Mix according to manufacturer's instructions. All amplifications were run on a authorized thermal cycler, the amplification conditions were one cycle at 94°C for 3 min, and then the PCR amplification was performed for 30 cycles, consisting of denaturation (94°C, 30 s), annealing [54°C, 1 min (or 55°C for GAPDH)], extension (72°C, 1 min), and an additional final extension step at 72°C for 5 min. P53 expression levels were determined using the following primers: 5′-GTTCCGAGAGCTGAATGAGG-3′ (forward) and 5′-TTTTATGGCGGGACGTAGAC-3′ (reverse); primers for GAPDH: 5′-CGTCTTCACCACCATGGAGA-3′ (forward) and 5′-CGGCCATCACGCCACAGTTT-3′ (reverse). The expected sizes for the PCR products: 125 bp for P53, 299 bp for GAPDH. The resulting PCR products were separated by electrophoresis in a 2%-(w/v)-agarose gel in TAE and stained with ethidium bromide. The mRNA levels were calculated as the ratios of optical density of the PCR products to that of the GAPDH PCR product using the program Image J.

### Statistical treatment

Data were presented as mean ± standard deviation (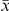
± s) , and results were analysed using SPSS16.0 software, significance was determined by one-way ANOVA.
